# Transcultural adaptation of the Johns Hopkins Fall Risk Assessment Tool

**DOI:** 10.1590/1518-8345.1158.2783

**Published:** 2016-08-29

**Authors:** Maria Carmen Martinez, Viviane Ernesto Iwamoto, Maria do Rosário Dias de Oliveira Latorre, Adriana Moreira Noronha, Ana Paula de Sousa Oliveira, Carlos Eduardo Alves Cardoso, Ifigenia Augusta Braga Marques, Patrícia Vendramim, Paula Cristina Lopes, Thais Helena Saes de Sant'Ana

**Affiliations:** 1 PhD, RN, WAF Informática, São Paulo, SP, Brazil.; 2 RN, Specialist in Surgical Center, Unidade de Internação, Hospital Samaritano de São Paulo, São Paulo, SP, Brazil.; 3 PhD, Full Professor, Faculdade de Saúde Pública, Universidade de São Paulo, São Paulo, SP, Brazil.; 4 RN, Unidade de Internação, Hospital Samaritano de São Paulo, São Paulo, SP, Brazil.; 5 Analist, Núcleo de Epidemiologia, Hospital Samaritano de São Paulo, São Paulo, SP, Brazil.; 6 RN, Specialist in Intensive Care and Cardiology, Unidade de Internação, Hospital Samaritano de São Paulo, São Paulo, SP, Brazil.; 7 RN, Pronto Socorro Infantil, Hospital Samaritano de São Paulo, São Paulo, SP, Brazil.; 8 Doctoral Student, Departamento de Enfermagem, Universidade Federal de São Paulo, São Paulo, SP, Brazil. Assistant Director, Divisão de Pneumologia, Instituto do Coração, Hospital das Clínicas, Faculdade de Medicina, Universidade de São Paulo, São Paulo, SP, Brazil.; 9 Coordinator, Núcleo de Oncologia, Núcleo de Oncologia, Hospital Samaritano de São Paulo, São Paulo, SP, Brazil.; 10 RN, Hospital Nove de Julho, São Paulo, SP, Brazil.

**Keywords:** Cross-Cultural Comparison, Validation Studies, Nursing Methodology Research, Accidental Falls, Quality of Health Care

## Abstract

**Objective:**

to perform the transcultural adaptation and content validity analysis of the Johns Hopkins Fall Risk Assessment Tool to assess both fall risk and fall-related injury risk for hospitalized elderly in Brazil.

**Method:**

the transcultural adaptation consisted of translating the scale to Portuguese (Brazil), back-translating it into its language of origin, establishing a consensus version, and having an expert committee verify its transcultural equivalence. Content assessment was conducted by a committee of judges, ending with the calculation of the items and scales' content validity index. Nurses tested the tool.

**Results:**

the scale's translated version went through two evaluation rounds by the judges, based on which, the items with unsatisfactory performance were changed. The content validity index for the items was ≥80.0% and the global index 97.1%. The experimental application showed the scale is user-friendly.

**Conclusion:**

the scale presents valid content for the assessment of fall risk and risk of fall-related injuries and is easy to use, with the potential to contribute to the proper identification of risks and the establishment of care actions.

## Introduction

Patient falls are defined as non-purposeful displacement of the body toward the ground, without timely correction, which may or may not be followed by injury to the patient or a lesion^1^ ^-^ ^3^. It is a frequent adverse event in hospitals and has a multifactor etiology. Its consequences may affect patients, family members, workers, hospitals and the health services' funding agencies^1^ ^-^ ^5^. 

Requirements regarding the quality and safety of care delivery both in Brazil and abroad have increased in recent years, which has driven the development and dissemination of better evidence-based practices^1^ ^-^ ^4^. Aware that falls are events that are sensitive to care practice, especially nursing care, hospitals and health facilities that are focused on the promotion of safe care emphasize the management of these events in the hospital setting^1^ ^-^ ^4^. The scope of research has expanded to encompass the development/improvement of resources and care strategies with the incorporation of economic analyses, emphasis on evaluation by using indicators, encouraging multidisciplinary interventions and promoting the implementation of protocols that systematize practices to assess risks and care approaches^1^ ^-^ ^6^.

The use of scales to assess risk is aligned with evidence-based practice to support the management of falls, emphasizing fall prevention and injury reduction^1^ ^-^ ^5^. Scales are generally composed of a set of items that represent risk factors, favoring the identification of patients and/or their classification in terms of levels of risk for the occurrence of falls^1^ ^,^ ^4^ ^,^ ^6^. It is also necessary to identify patients at the risk of suffering further damage caused by a fall, aiming to prevent severe injuries in more susceptible patients^1^ ^,^ ^5^.

Tools intended to assess risks should be valid for the target populations, otherwise they may generate bias or lead to identification/classification errors^4^ ^,^ ^6^. There are currently approximately 50 instruments to assess fall risk reported in the international literature^6^ ^-^ ^7^; however, there are no instruments developed and/or validated in Brazil. An exception is the Morse Fall Scale, which was submitted to a transcultural adaptation to be used in Brazil. The accuracy of its Brazilian version, though, has not been analyzed yet^8^, and even this scale, already transculturally adapted, does not identify patients at high risk for fall-related injuries.

The use of these scales is limited due to issues related to the methods used to develop and validate them, such as: lack of clear criteria to establish the tool's content; gaps in the tool's structure that fail to comprise the main aspects of the construct under analysis; the inclusion of risk factors without consistent predictive value; lack of longitudinal studies, which compromises the assessment of causal relationships between risk and event; inappropriate samples; inappropriate statistics and/or the failure to include control variables; subjective criteria for scoring or classifying subjective scores; and being restricted to a specific population^6^ ^-^ ^7^. There are also restrictions of an operational nature, considering that a scale should have low cost and be easily and quickly applied^9^.

Considering these aspects and the need to include a valid method to assess fall risk in health services, this study's authors conducted a search in the literature to find such tools and the Johns Hopkins Fall Risk Assessment Tool - JH-FRAT was identified. This scale has proven to be simple, with a relatively low cost (use fee), with a history of application in a context of managed healthcare and with established content validity^10^ ^-^ ^11^. 

Considering the importance of assessing risks in the context of care delivery, the relevance of tools' measurement abilities, and a lack of tools properly adapted and validated in Brazil, this study is intended to present the transcultural adaptation and content validity analysis of the JH-FRAT to assess fall risk and risk for fall-related injuries among hospitalized adult patients. 

## Method

### Study design 

This methodological study was developed in two phases: transcultural adaptation and assessment of content validity of the Johns Hopkins Fall Risk Assessment Tool - JH-FRAT *(Escala de avaliação de risco de queda Johns Hopkins - JH-FRAT*) to be used in Brazil, conducted in 2014 in a high complexity care hospital in the city of São Paulo, SP, Brazil.

### Tool of interest

JH-FRAT was designed by professionals and researchers from the Johns Hopkins Hospital and Johns Hopkins University, School of Nursing, in the context of the facility's management of falls. It has a patient-centered approach aimed to prevent falls and fall-related injuries^10^. To structure the scale, the tools available in the literature were first analyzed, classifying evidence related to risk identifiers and giving priority to those that were significantly correlated to falls. A risk classification was assigned to each factor (low, moderate, high), which was established to guide preventive measures. Based on the assessed tools, a score model for the scale risk was proposed. This model was tested in different contexts of patients and adjusted by a consensus group^10^. 

After two years of using the JH-FRAT in the facility, its acceptability and content validity were assessed by an expert committee composed of the researchers who developed the scale and professionals who used it^11^. Content analysis assessed the scale's structure and its items, the relationship between each item and the composition of final risk assessment, as well as the tool's scoring model. Additionally, nurses who used the instrument assessed clarity and interpretation of the scale's items, relevance of each item to assess fall risk and the semantic interpretation of each item. As a result of these assessments, redaction, scoring and score composition were revised^11^.

In the end, the scale was composed of 8 aspects of fall risk: (1) prior situations that defined risk: immobilization, prior history of falls, history of falls during hospitalization, and patient considered to be at high risk according to protocols; (2) age; (3) history of falls; (4) eliminations; (5) medication; (6) assistive devices; (7) mobility; (8) cognition. Total score ranges from 0 to 35 points and can be characterized into low, moderate and high risk^10^ ^-^ ^11^.

The studies addressing the JH-FRAT's measurement properties report valid content and acceptable sensitivity and specificity, though there are some methodological limitations that hinder the generalization of results^10^ ^-^ ^12^. The authors still need to conduct further studies^10^ ^-^ ^11^.

### Transcultural adaptation

The transcultural adaptation was based on a framework proposed in the literature^13^ emphasizing semantic (meaning of words and grammatical aspects), idiomatic (adapting colloquial expressions to equivalent ones from the target language), experiential (expressions that portray cultural experiences or situations without correspondence in the target culture), and conceptual (similar words with conceptual differences between cultures) equivalence. 

-Stage 1 - initial translation: was performed by two independent Brazilian translators. According to the proposal of the adopted theoretical framework^13^, one of the translators was aware of the objectives, concepts and content underlying the material to be translated, providing a more critical perspective of the translation. The second translator had no prior knowledge, which favored the identification of different meanings of the original content. Both received instructions on performing the translation, the original version JH-FRAT and a spreadsheet to record the translation of each item.

-Stage 2 - synthesis of translations: the two translators and two researchers reached a reconciled version of both translations through discussion and consensus, emphasizing the four aspects of equivalence previously mentioned. This reconciled version was accompanied by a report that records the process and the solutions adopted when discrepancies appeared. 

-Stage 3 - back-translation: the synthesized version was back-translated by a sworn bilingual translator, who is a native speaker in the scale's language of origin, and was unaware of the concepts adopted. The translator received correspondence containing orientation regarding the back-translation, the translated synthesis version and a spreadsheet to record the back-translation of each item. 

-Stage 4 - Expert committee: it was composed of six members of the research project, who were methodologists and/or healthcare workers and/or mastered the languages. Their role was to consolidate a single, pre-final version of the previous versions (original version, translated versions, consensus version, and back-translation). 

A structured script was used and the opinion of each expert regarding the transcultural adaptation of each item was recorded into three categories of response (appropriate, partially appropriate, inappropriate). Items that obtained agreement greater than 90.0% among experts in the category "appropriate" were considered satisfactory^14^. The reasons for the non-appropriateness of items that obtained agreement less than 90.0% were discussed, as well as how they could be improved, until consensus was reached. The remaining items were also analyzed in order to identify aspects that could be improved.

-Stage 5 - pre-final version test: at the end of the process, as described in "Stage 3", content validity was assessed. 

### Assessment of content validity

Assessing content validity consists of a judgment in which experts verify whether items that compose the instrument represent what is actually intended to be measured^15^ ^-^ ^16^. For that, the assessment of content validity was conducted in two stages:

-Stage 1 - Committee of judges: composed of 6 professionals with acknowledged expertise, mastery, and proficiency in the subject areas underlying the constructs. A document was developed to present the conceptual definition of constructs (risk for falls and high risk for fall-related injuries), the study's objectives, and instructions to complete the assessment script, attached to the original version and to the pre-final translated version.

A presentation document along with a script was used to record the opinions of each expert in regard to the validity of each item. The scale was split into 34 evaluation items, including title, instructions, questions, and categories of options of responses. Based on previous studies^14^ ^,^ ^16^, each item was assessed into categories of responses (1 - Invalid without possibility of revision; 2 - Partially invalid, requires review; 3 - Partially valid, requires minor review; 4 - Valid), considering criteria such as objectivity (clear and accurate redaction), relevance (item represents the attribute under analysis and does not imply divergent attributes), simplicity (item expresses a single idea), accuracy (item does not allow for confusion or repetition in regard to other items), and accessibility (the assessment is rapidly applied, with minimum effort, time and resources). In addition to the assessment of each item, three questions were added in regard to the scale's general assessment: (a) absence of any item that is relevant for the construct; (b) representation of the construct "high risk of fall-related injury" in items 3 to 5; (c) representation of the construct "fall risk" as a whole. The items that obtained a score of 4 - Valid, were considered appropriate.

-Stage 2 - Analysis of agreement among judges: according to recommendations provided in the literature^14^, the following estimates were computed:

-I-CVI - Content validity index for items: percentage of judges who agreed with the item's adaptation (score of 4). Item with agreement greater than 80.0% were considered valid^14^. I-CVI was also estimated for each of the 3 questions concerning general appreciation for the scale.

-S-CVI - Content validity index for scale: it consists of the average of the items' performance (results of each I-CVI is totaled and divided by the total of items assessed). The cut-off point to consider the scale valid in terms of content was 90.0% agreement^14^.

Afterwards, a meeting was held with the researchers to discuss why items were considered inappropriate and how they could be improved until consensus was reached and a new pre-final version was established. Because some items were subject to controversy and obtained less than 80.0% validity among the judges, we opted to send the reformulated scale for the judges to assess a second time.

Stage 3 - Pre-final version test: the pre-test consisted of an experimental application of the scale's pre-final version with individuals from the target population (nurses who will use the scale). This stage was intended to verify whether the adapted version retained its equivalence in the context of a real application^13^. Twelve nurses from hospitalization units (one from each sector) who were selected by the units' heads, were invited to participate. Each nurse assessed the fall risk of 3 adult inpatients from the hospital under study.

A brief script was used to assess the 8 items that compose JH-FRAT and the risk-scoring format in which each nurse displayed their opinions regarding the validity of items using a Likert scale ranging from 1 (invalid, without possibility of review) to 4 (valid). This script also recorded the time (minutes) used to apply the scale for each patient. A descriptive analysis regarding the scores obtained was conducted (mean and standard deviation). The project was approved by the Institutional Review Board at Samaritano Hospital (report No. 678,566 de 08/06/2014) and complied with the principles of the Declaration of Helsinki.

The authors of JH-FRAT authorized the study and approved its use. Minor modifications required during the transcultural adaptation and content validity assessment were submitted for the authors' consideration. The consent of the scale's authors is required both for academic purposes and for the scale to be used in services.

## Results

### Transcultural adaptation

The reconciled version that resulted from the two translations and back-translation was assessed by the expert committee, which verified the transcultural equivalence of each item. Most items obtained 100.0% of agreement among experts. Improvements were proposed for the items that did not show satisfactory performance: 

Item 5 - "*Patient is deemed high fall-risk per protocol (e.g., seizure precautions). Implement high fall-risk interventions per protocol*": 0.0% agreement regarding its equivalence. The experts pointed out that the example provided in the reconciled version could be confusing in the case of hospitals with different protocols. Therefore, we opted to exclude the example and not specify protocols. 

Item 6 - "*Complete the following and calculate fall risk score. If no box is checked, score for category is 0*": 0.0% agreement in regard to the item's equivalence. The item's second phrase left room for doubt: it was unclear whether it referred to the general score or per category. Thus, we opted to revise the redaction to facilitate understanding.

Item 11 - "G*reater than or equal to 80 years (3 points*)": 0.0% agreement was obtained regarding the item's equivalence. "Greater than or equal to 80 years" was replaced by "80 years old or older" because it is a more colloquial expression. 

Item 18 - "*Medications: Include PCA/opiates, anti-convulsants, anti-hypertensives, diuretics, hypnotics, laxatives, sedatives, and psychotropics (Single-Select)*": 0.0% agreement in regard to its equivalence. Terms more usually used in nursing notation were adopted and the acronym PCA was spelled out to facilitate understanding of professionals not familiar with this resource

The pre-final version was established after items were reviewed. It was then submitted to content validity. 

### Content validity assessment 

Table 1 presents each item in its original version (English) and the I-CVI of the 1^st^ and 2^nd^ rounds. In the 1^st^ round, the judges pointed out situations they considered inappropriate and also made suggestions. These situations essentially referred to the redaction of phrases or the presence of technical terms, the translations of which could not generate misunderstandings. S-CVI was 80.9%. After the suggested adaptations were incorporated into the scale, a second round was held with the committee of judges, in which all items obtained I-CVI ≥ 80.0% and S-CVI was 97.1%, showing that each item was isolated and the scale as a whole presented appropriate content validity. For the three questions related to the scale's generic assessment, I-CVI was 100.0%; that is, the committee of judges unanimously considered that: (a) all the items/dimensions relevant for the construct "fall risk" were included in the scale; (b) items 3, 4 and 5 were sufficient and representative of the construct "high risk of fall-related injury"; and (c) in general, the scale presented valid content to assess the construct "fall risk". 

**Table 1:** Johns Hopkins Fall Risk Assessment Tool - JH-FRAT: results concerning I-CVI* of each item in two rounds of the assessment conducted by the committee of judges, São Paulo - SP - Brazil, 2014

Item	Content	Round 1	Round 2
0	The Johns Hopkins Fall Risk Assessment Tool - JH-FRAT	100.0	100.0
1	Fall Risk Factor Category: Scoring not completed for the following reason(s) (check any that apply). Enter risk category (i.e., Low/High) based on box selected.	50.0	100.0
2	·Complete paralysis, or completely immobilized. Implement basic safety (low fall risk) interventions.	66.7	100.0
3	Patient has a history of more than one fall in 6 months prior to admission. Implement high fall risk interventions throughout hospitalization.	83.3	100.0
4	Patient has experienced a fall during current hospitalization. Implement high fall risk interventions throughout.	66.7	100.0
5	Patient is deemed high fall-risk per protocol (e.g., seizure precautions). Implement high fall-risk interventions per protocol.	66.7	100.0
6	Complete the following and calculate fall risk score. If no box is checked, score for category is 0.	100.0	100.0
7	Age (Single-Select).	100.0	100.0
8	60 - 69 years (1 point).	100.0	100.0
9	70 - 79 years (2 points).	100.0	100.0
10	Greater than or equal to 80 years (3 points).	100.0	100.0
11	Fall History (Single-Select).	50.0	100.0
12	One fall within 6 months before admission (5 points).	66.7	100.0
13	Elimination. Bowel and Urine (Single-Select).	100.0	100.0
14	Incontinence (2 points).	83.3	80.0
15	Urgency or frequency (2 points).	66.7	100.0
16	Urgency/frequency and incontinence (4 points).	66.7	100.0
17	Medications: Include PCA/opiates, anti-convulsants, anti-hypertensives, diuretics, hypnotics, laxatives, sedatives, and psychotropics (Single-Select).	66.7	100.0
18	On 1 high fall risk drug (3 points).	66.7	100.0
19	On 2 or more high fall risk drugs (5 points).	66.7	100.0
20	Sedated procedure within past 24 hours (7 points).	50.0	100.0
21	Patient Care Equipment: Any Equipment That Tethers Patient (e.g., IV Infusion, Chest Tube, Indwelling Catheters, SCDs, etc.) (Single-Select).	83.3	80.0
22	One present (1 point).	100.0	80.0
23	Two present (2 points).	100.0	80,0
24	3 or more present (3 points).	100.0	80.0
25	Mobility (Multi-select, Choose all that apply and add points together).	100.0	100.0
26	equires assistance or supervision for mobility, transfer, or ambulation (2 points).	83.3	100.0
27	Unsteady gait (2 points).	83.3	100.0
28	Visual or auditory impairment affecting mobility (2 points).	83.3	100.0
29	Cognition (Multi-select, Choose all that apply and add points together).	100.0	100.0
30	Altered awareness of immediate physical environment (1 point).	83.3	100.0
31	Impulsive (2 points).	50.0	100.0
32	Lack of understanding of one's physical and cognitive limitations (4 points).	100.0	100.0
33	*Moderate risk = 6-13 Total Points, High risk > 13 Total Points	66.7	100.0

*I-CVI - Content validity index for items.

It should be noted that, similar to the experts committee, the committee of judges also questioned item 5 - "*Patient is deemed high fall-risk per protocol (e.g., seizure precautions). Implement high fall-risk interventions per protocol*," especially in regard to the understanding of "protocols". Redaction was changed again and new examples were included, not specifying which situations were subject to protocols, since health facilities in Brazil are in different stages regarding the management of care according to protocols. 

All items analyzed in the pre-test obtained means greater than 3.5 points (scale from 1 to 4 points) and the mean score of the instrument as a whole was 3.8 points, showing the that nurses considered the Brazilian version of JH-FRAT to be valid for assessing fall risk and no other changes were necessary. The results are presented in Table 2. The nurses reported they spent 3.0 minutes (SD=1.5), on average, to apply the scale to each patient.

**Table 2:** Johns Hopkins Fall Risk Assessment Tool - JH-FRAT: score obtained in the pre-test of the final version adapted to be used in Brazil, São Paulo - SP - Brazil, 2014

Item assessed	Mean*(SD)^†^
1) First block with instructions and situations that pre-defined risk situations	3.8 (0.5)
2) Risk factor "age"	4.0 (0.0)
3) Risk factor "history of falls"	3.8 (0.9)
4) Risk factor "eliminations"	3.8 (0.6)
5) Risk factor "use of medication with high fall risk"	3.8 (0.6)
6) Risk factor "assistive devices"	4.0 (0.0)
7) Risk factor "mobility"	3.9 (0.3)
8) Risk factor "cognition"	3.6 (0.7)
Set of items	3.8 (0.3)

* Score from 1 to 4 points † SD = standard deviation

**Figure 1:**
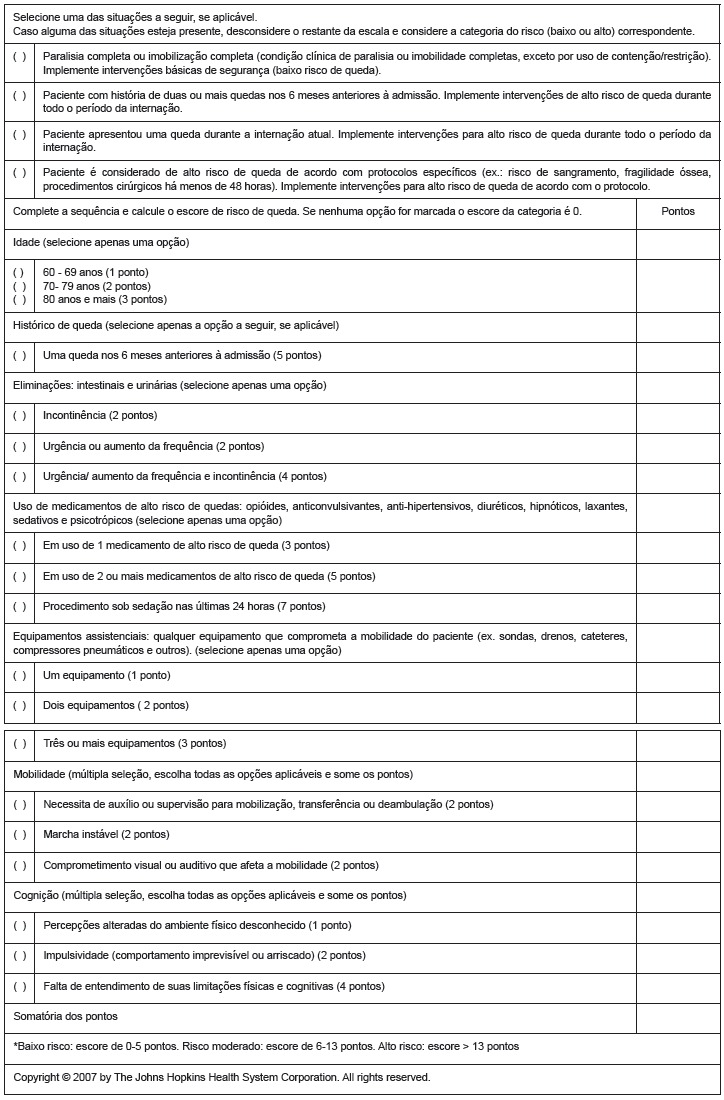
Adapted version of Johns Hopkins Fall Risk Assessment Tool to be used in Brazil

Given the favorable results from the pre-test, the scale was considered to have content validity. Figure 1 presents the final version, adapted and validated for use in Brazil, with all the adaptations incorporated during the process.

## Discussion

This study's objective was to present the process of transcultural adaptation and content validation of JH-FRAT. The results show this instrument presents valid content to assess risk for falls and risk for fall-related injury in hospitalized adult patients. 

Some items did not present satisfactory performance in the transcultural adaptation process and this situation was especially true when items provided examples of protocols or technical terms, the translation of which had the potential to generate doubts and compromise understanding. Because Brazilian health facilities are in different stages of care quality and safety management, the redaction was revised in order to retain flexibility and align the scale to the needs of each local context. Unfortunately, there are no studies addressing the transcultural adaptation of JH-FRAT in other populations, which limits comparison of results obtained in this stage or the solutions adopted.

The content validity assessment analyzes the representativeness or appropriateness of the instrument's content; that is, it verifies whether it represents what it is intended to measure, comprising the construct's different aspects^15^. The committee of judges responsible for making this assessment was composed of nursing professionals external to the facility where the study was conducted whose competence in the subject and method underlying the study was recognized. The committee from the original study was also composed of individuals with combined experience and knowledge in clinical care, management, quality improvement, and patient education^11^.

In the original study, the committee assessed the appropriateness of each of the scale's 8 aspects of risk to measure fall risk, as well as the appropriateness of each of the response options for each aspect of risk. As a result, the authors implemented minor changes in the scale, reaching the final version^11^. This is the version submitted to content validation in this study and, in addition to adaptation of each aspect, the analysis was deepened to assess whether the construct "fall risk" and the construct "high risk of fall-related injury" are represented. Similar to the original study, this study showed the need to make minor corrections in the scale's redaction.

Even though there are different understandings regarding a instrument's content validity, we believe that the assessment process conducted by a committee of judges regarding the content of items, coupled with an analysis of quantitative agreement among judges and a pre-test, demonstrate the content validity of the adapted instrument to another culture^13^ ^-^ ^16^. In this study, after the committee of judges assessed the instrument and obtained satisfactory I-CVI and S-CVI, a pre-test was conducted.

The pre-test showed that the scale's adapted version retained its equivalence when applied to a real context, assessing the perceptions of nurses regarding the validity or each of the eight aspects of risk. The pre-test applied in the original study was also conducted with nurses who would use the scale in real situations in different clinical fields, assessing the clarity and interpretation of items, to what extent each item was consistent with the assessment of fall risk, and whether different groups of patients assessed specific items differently^11^. The results presented initial evidence of satisfactory content validity and guided the adaptation process, favoring greater clarity and consistency among different hospital settings^11^. 

Ideally, a scale should possess the following characteristics: rapid application, ease of understanding, low cost, and cause no harm/inconvenience^6^ ^,^ ^9^. The pre-test also showed that the JH-FRAT meets these requirements. Despite this simplicity, there is a need to provide detailed instructions and training to professionals who will use this tool. 

Another aspect to be considered is the regularity with which the scale can be applied. The scale's authors indicate that assessment is performed at the time the patient is admitted into the unit or sector and once a day and whenever the patient's clinical conditions or treatment changes^17^. Opportunities to assess fall risk are relevant, regardless of what scale is used.

The scale's authors indicate that the initial assessment should be performed by Registered Nurses and that Licensed Practical Nurses (the equivalent in Brazil would be nursing technicians and auxiliaries) could perform subsequent assessments^17^. In Brazil, however, considering the Nursing Professional Practice Law^18^ and the educational background of those composing the nursing staff, this assessment should be a competence exclusive to nurses. 

Authorization to use JH-FRAT's adapted version in Brazil: facilities/professionals who desire to apply the scale should ask for a license at the Institute for Johns Hopkins Nursing - IJHN (https://www.ijhn-education.org/content/johns-hopkins-fall-risk-assessment-tool). The authors of this transcultural adaptation do not provide licenses nor do they have any share in the amounts paid to IJHN. 

JH-FRAT is a tool to guide care and does not replace a nurse's clinical judgment. This issue is relevant because even properly constructed scales may fail to predict events, and more importantly than classifying the risk of patients, proper individualized care for managing the risks to each patient is revealed^4^ ^,^ ^6^. Additionally, the assessment of fall risk should take place in the context of managed care, inserted in quality of care management programs^1^ ^,^ ^4^. Its application outside of this context may not result in expected benefits. An instrument used to assess risk should be valid for the purpose of what it was originally designed to avoid identification/classification of risk errors^4^ ^,^ ^6^ ^,^ ^15^. In this sense, content validity assessment is only one of the steps toward validation, so that the need to add other assessments, such as measure properties, including those that assess reliability and accuracy^13^ ^,^ ^15^, remain. Therefore, researchers are conducting a longitudinal study to assess other properties of the measure and cut-off points of the JH-FRAT Brazilian version. It is worth noting that this study was focused on the application of the scale among elderly inpatients, thus its application within other population groups should be carefully considered.

 It is worth noting that we are finalizing a longitudinal quantitative study assessing the tool's construct and criterion validity, in addition to accuracy.

## Conclusions

The transcultural adaptation processes and content validity assessment sought to include the primary stages necessary to provide a valid version of the JH-FRAT to be used in Brazil. The process included two committees of qualified professionals. The first committee (experts) emphasized the transcultural equivalence (semantic, idiomatic, experiential, and conceptual), while the second committee (judges) emphasized the content appropriateness (structure and composition) of the instrument to represent the construct it is supposed to measure. The problems identified were basically related to grammatical aspects, conceptualization and redaction of items, which led to the need for corrections and improvements. No problems regarding the content of the translated versions were found. The pre-test was conducted with nurses considered to represent the target population. The nurses considered the translated and adapted version to be valid and easy to apply in the health care service. 

The results show the validity of the translated scale, with structure focused on the assessment of fall risk and the risk of fall-related injuries, indicating it is appropriate and aligned with processes of managed care practice.

A limitation of this study is the measurement proprieties of the JH-FRAT. Only the analysis of content validity was performed, while other types of analysis were not addressed. Another aspect is that this study was conducted in a specific context (hospital facility of high complexity in the city of São Paulo). Even though the researchers, experts and judges were concerned with idiomatic breadth, further studies conducted in other hospital facilities should be conducted.

Finally, this transcultural adaptation study and assessment of content validity of the JH-FRAT to be used in Brazil showed that the translated version has valid content to assess "fall risk" and also "high risk of fall-related injuries". The scale is easily and rapidly applied, which contributes to proper identification of risks and implementation of care actions.
